# A case of Trousseau’s syndrome due to intrahepatic cholangiocarcinoma with an extremely high level of CA19-9

**DOI:** 10.1186/s40792-020-00835-8

**Published:** 2020-04-19

**Authors:** Ryosuke Sasaki, Yuki Ohya, Shintaro Hayashida, Yuto Maeda, Shuei Murahashi, Sayahito Kumamoto, Akira Tsuji, Hidekatsu Shibata, Kunitaka Kuramoto, Hironori Hayashi, Kazumi Kuriwaki, Masayoshi Iizaka, Osamu Nakahara, Yukihiro Inomata

**Affiliations:** 1 Department of Surgery, Kumamoto Rosai Hospital, 1670 Takehara-machi, Yatsushiro, Kumamoto, 866-8533 Japan; 2 Department of Neurology, Kumamoto Rosai Hospital, 1670 Takehara-machi, Yatsushiro, Kumamoto, 866-8533 Japan; 3 Department of Diagnostic Pathology, Kumamoto Rosai Hospital, 1670 Takehara-machi, Yatsushiro, Kumamoto, 866-8533 Japan

**Keywords:** Trousseau’s syndrome, Intrahepatic cholangiocarcinoma, Carbohydrate antigen 19-9

## Abstract

**Background:**

Trousseau’s syndrome is a cancer-associated thrombosis. Trousseau’s syndrome with cholangiocarcinoma is a rare condition with poor prognosis.

**Case presentation:**

A 59-year-old female was admitted to our hospital with abdominal pain, headache, and nausea. Abdominal enhanced computed tomography revealed liver tumor, splenic infarction, and bilateral renal infarction. Multiple acute cerebral infarctions were also detected by magnetic resonance imaging. Her preoperative serum levels of carbohydrate antigen 19-9 (CA19-9) and carcinoembryonic antigen (CEA) were > 120,000 U/mL and 589.6 ng/mL, respectively, which were extremely high. Histopathology after right hepatectomy revealed moderately differentiated adenocarcinoma consistent with intrahepatic cholangiocarcinoma. Her serum levels of CA19-9 were trending down to 9029.2 and 2659.8 U/mL at 1 and 3 weeks after surgery, respectively. However, at 7 weeks after surgery, her CA19-9 levels increased in the presence of positive imaging findings in the remnant liver, hilar lymph nodes, and peritoneal cavity. The initiation of combination chemotherapy including gemcitabine and cisplatin had a significant effect. The patient was doing well at 6 months after the surgery.

**Conclusion:**

This rare case of Trousseau’s syndrome due to cholangiocarcinoma suggests that extremely high CA19-9 levels might be a pathogenic factor of this syndrome.

## Background

Armand Trousseau was the first to describe the association of thrombotic episodes with an underlying malignancy [1–5]. Recent definitions restrict Trousseau’s syndrome to unexplained thrombotic events that occur before or concomitantly with the presentation of visceral malignancies [3]. The prognosis of Trousseau’s syndrome, which is often diagnosed in patients with terminal cancer, is extremely poor. While the precise mechanism underlying Trousseau’s syndrome remains unknown, hypercoagulability might be initiated by mucins produced by an adenocarcinoma [4]. Intriguingly, Trousseau’s syndrome is rarely reported in patients with cholangiocarcinoma [3]. We herein report the case of a patient with intrahepatic cholangiocarcinoma who presented with Trousseau’s syndrome manifesting as thromboembolic lesions in multiple organs including the brain and was treated with right hepatectomy followed by systemic chemotherapy.

## Case presentation

**Fig. 1:**
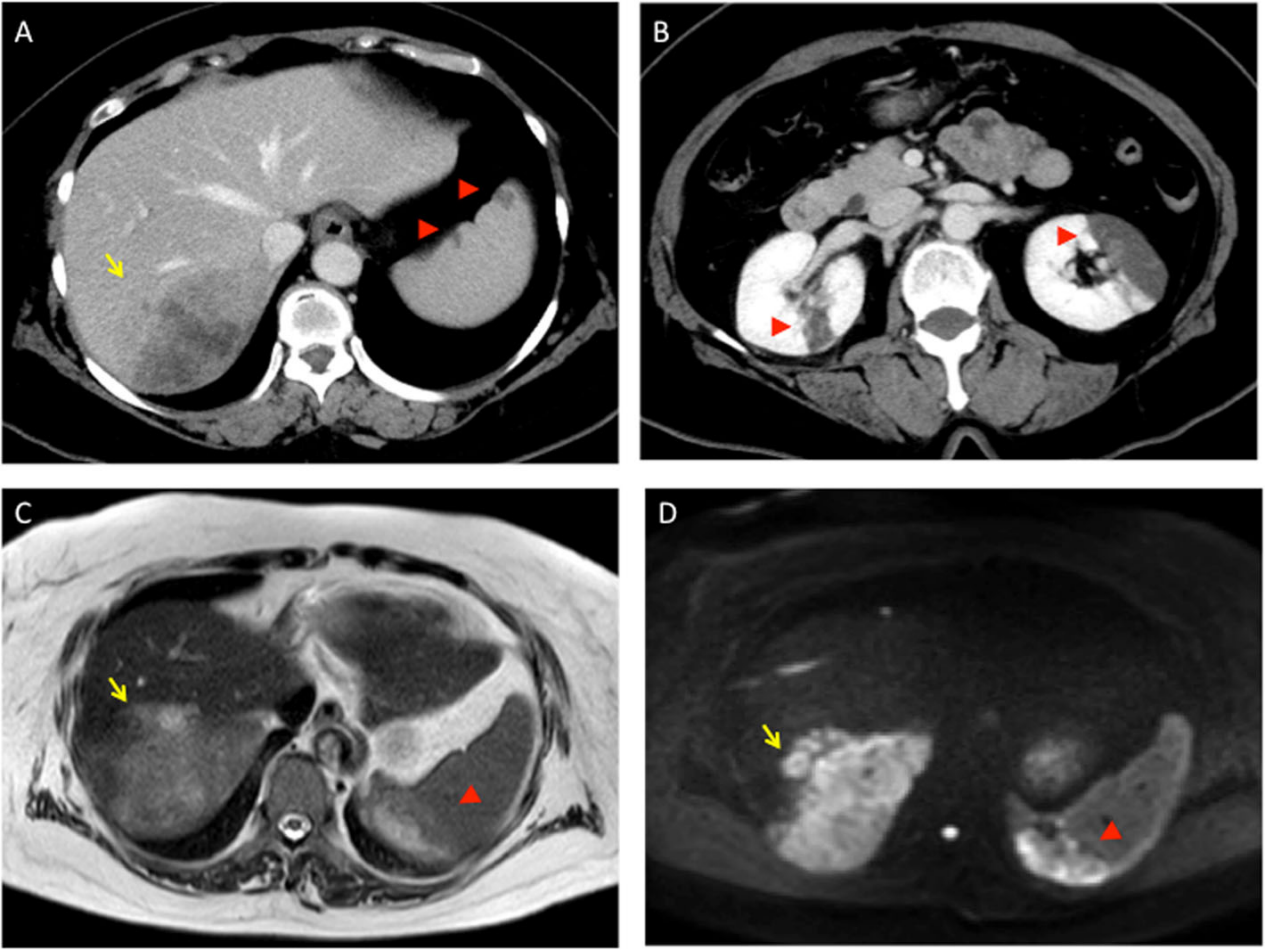
Abdominal enhanced CT and MRI findings. **a** CT showing a tumor in the posterior segment of the right hepatic lobe (arrow) and splenic infarctions (arrowhead). **b** CT showing renal infarctions in both kidneys (arrowhead). **c**, **d** MRI showing a tumor in the posterior segment of the right hepatic lobe (arrow) and splenic infarctions (arrowhead). CT, computed tomography; MRI, magnetic resonance imaging

A 59-year-old female visited an emergency room with the chief complaints of abdominal pain and dizziness and was diagnosed with gastroenteritis and vertigo. Three days later, she was readmitted to the emergency room because of worsening abdominal pain and difficulty in movement due to headache and nausea. Plain whole-body computed tomography (CT) showed suspicious cerebral infarction and a low-density mass lesion in right liver lobe. Subsequent head magnetic resonance imaging (MRI) confirmed infarcted areas in multiple blood vessels defined as an embolic shower. Contrast-enhanced CT revealed about 9 × 6 × 4 cm-sized and ill-defined mass with several daughter nodules in the posterior segment of the right hepatic lobe, splenic infarction, and bilateral renal infarctions (Fig. 1). The liver hypodense mass had a peripheral rim-like enhancement during the arterial phase and contacted with diaphragm, retroperitoneum, and inferior vena cava. The tumor in the liver was diagnosed as intrahepatic mass-forming cholangiocarcinoma. There was no suspicion of other gastrointestinal or gynecological malignancies. At that time of diagnosis, the level of carbohydrate antigen 19-9 (CA19-9) was > 120,000 U/mL, above the detectable range, and the level of carcinoembryonic antigen (CEA) was 211.7 ng/mL. The coagulation profile of the patient was as follows: platelet count, 8.7 × 10^4^/μL; D-dimer, 36.6 μg/mL; prothrombin time-international normalized ratio, 1.16; and activated partial thromboplastin time, 28.1 s. Ultrasonography of the carotid arteries and lower limbs, transthoracic echocardiography, and Holter electrocardiogram did not reveal the source of embolism. Therefore, the patient was diagnosed with Trousseau’s syndrome due to intrahepatic cholangiocarcinoma.

Continuous intravenous infusion of unfractionated heparin at a dose of 10,000 U per day was started immediately. However, she developed left hemiplegia 1 week later, and head MRI revealed thrombosis of the right middle cerebral artery. Activated partial thromboplastin time value was 23.4 s at that time in spite of continuous administration of heparin. She was transferred to a hospital where endovascular treatment was possible, and thrombectomy was performed. Although left paralysis remained after thrombectomy, she returned to our hospital for the treatment of intrahepatic cholangiocarcinoma. In the meantime, she was treated with continuous infusion of unfractionated heparin at a dose of 15,000 U per day. After the thrombectomy, activated partial thromboplastin time value was kept nearly 40.0 s with administration of heparin.

Preoperative examination did not show any issues with cardiopulmonary function. Abdominal enhanced CT and MRI showed a tumor in the posterior segment of the right hepatic lobe (Fig. 1). Hepatic reserve after right hepatectomy was confirmed with indocyanine green clearance test and technetium-99m diethylenetriamine penta-acetic acid-galactosyl human serum albumin (99mTc-GSA) liver scintigraphy (data not shown). Although the preoperative serum levels of CA19-9 and CEA were extremely high, the preoperative D-dimer, fibrin degradation product (FDP), and fibrinogen levels were slightly higher than the normal limits (Table 1). Protein C level was 154% (normal 70–150), protein S level was 164% (normal 65–135), and cardiolipin antibodies were normal (IgG, < 8; IgM, 5).

**Table 1:** Tumor maker levels and coagulation tests

[normal range]	Pre-operative TMs and CTs	TMs and CTs at POW4
CA19-9 (U/mL) [0.0-37.0]	>12,0000	4905.5
CEA (ng/mL) [0.0-5.0]	589.6	6.3
CA125 (U/mL) [0.0-35.0]	64.8	104.8
CA15-3 (U/mL) [0.0-31.3]	95.4	15.3
SLX (U/mL) [0.0-38.0]	330	46
D-dimer (mg/mL) [0.0-1.0]	6.2	3.2
FDP (mg/mL) [0.0-4.9]	8.5	6.2
fibrinogen (mg/dL) [200-400]	438	272

*TM* tumor maker, *CT* coagulation test, *POW4* postoperative week 4, *CA19-9* carbohydrate antigen 19-9, *CEA* carcinoembryonic antigen, *CA125* cancer antigen 125, *CA15-3* Cancer antigen 15-3, *SLX* Sialyl Lewis X, *FDP* fibrin degradation product

During the surgery, anticoagulation was performed with subcutaneous injection of low-molecular-weight heparin at a dose of 5000 U per 12 h. In surgery, the tumor was exposed on the liver surface and partially adhered to the diaphragm and retroperitoneum. Right hepatectomy was performed with combined resection of the invading tumor.

**Fig. 2:**
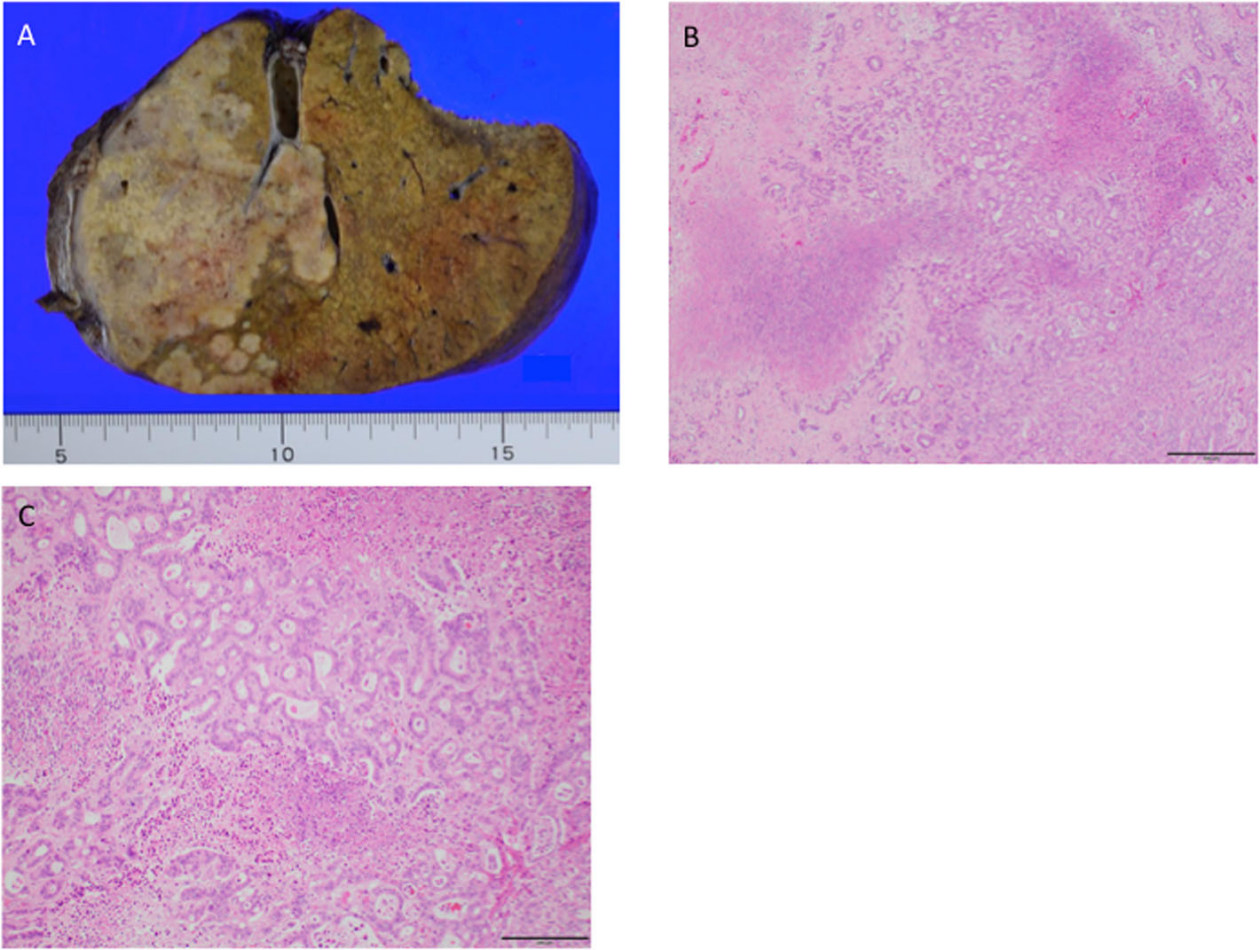
Intrahepatic cholangiocarcinoma. **a** Cut surface of the formalin-fixed liver with solid masses in the right lobe. **b** Hematoxylin and eosin staining. Magnification, × 40. Scale bar, 500 μm. **c** Hematoxylin and eosin staining. Magnification, × 100. Scale bar, 200 μm

The resected tumor was 9 × 6 × 6 cm in size, and the pathological diagnosis was moderately differentiated intrahepatic cholangiocarcinoma (pT4pN0M0 pStage IVA, vp1, vv2, b1, UICC8th) (Fig. 2). Intraoperative irrigation cytology was negative, and lymph nodes of no.12 did not show metastasis. Tumor invasion to the resected diaphragm was pathologically confirmed, but there was no invasion to the retroperitoneum.

**Fig. 3:**
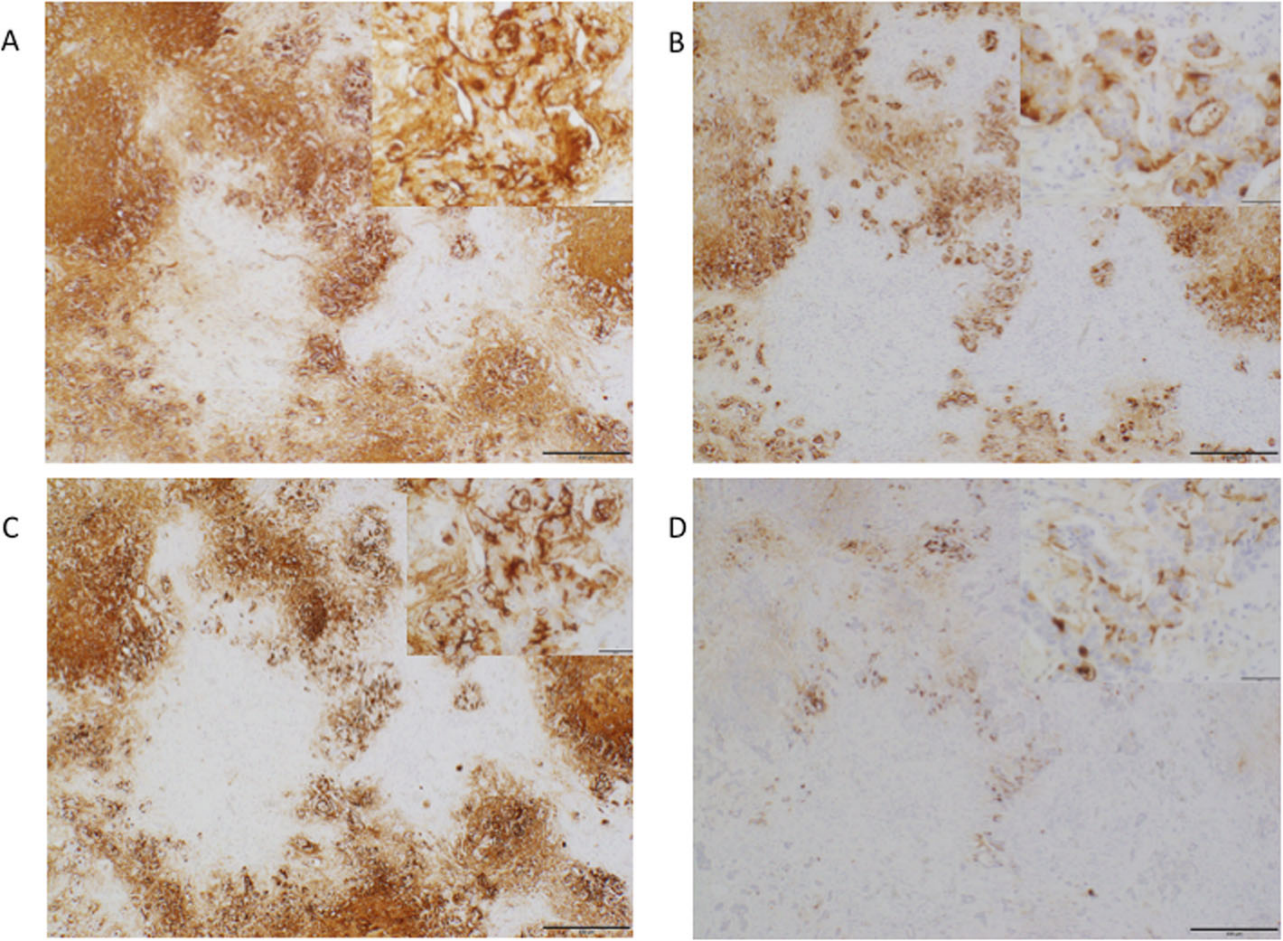
Immunohistochemical findings. Immunohistochemistry for CA19-9 (**a**), CEA (**b**), MUC-1 (**c**), and CA125 (**d**). Magnification, × 40 and × 200. Note strong expression of CA19-9, CEA, and MUC-1 in the cytoplasm and membrane of cholangiocarcinoma cells. CA125 expression in the cytoplasm of cholangiocarcinoma cells is weak and partial. Scale bar 500 μm and 50 μm. CA19-9, carbohydrate antigen 19-9; CEA, carcinoembryonic antigen; MUC-1, cell surface-associated mucin 1; CA125, cancer antigen 125

Immunohistochemistry was performed for cancer antigen 125 (CA125; clone M3519; Dako, Glostrup, Denmark), CEA (clone M7072; Dako), cell surface-associated mucin 1 recognized by the cancer antigen 15-3 (CA15-3) epitope (MUC-1; clone MA552; Leica Biosystems, Nussloch, Germany), and CA19-9 (M3517; Dako). As seen in Fig. 3, strong expression levels of CA19-9, CEA, and MUC-1 were observed in the cytoplasm and membrane of cholangiocarcinoma cells.

**Fig. 4:**
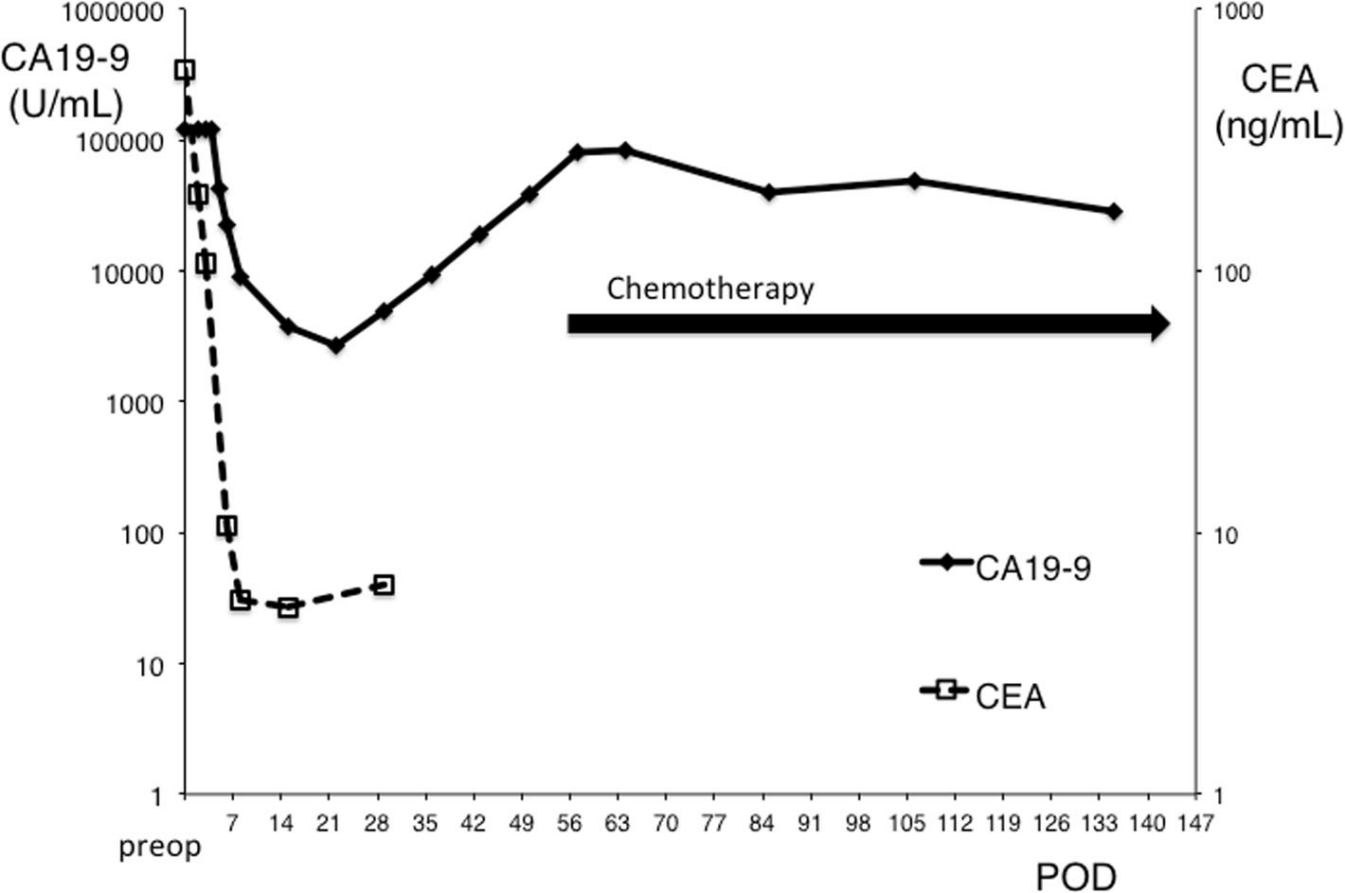
Perioperative changes in CA19-9 and CEA levels. Carbohydrate antigen 19-9; CEA, carcinoembryonic antigen; POD, postoperative day

**Fig. 5:**
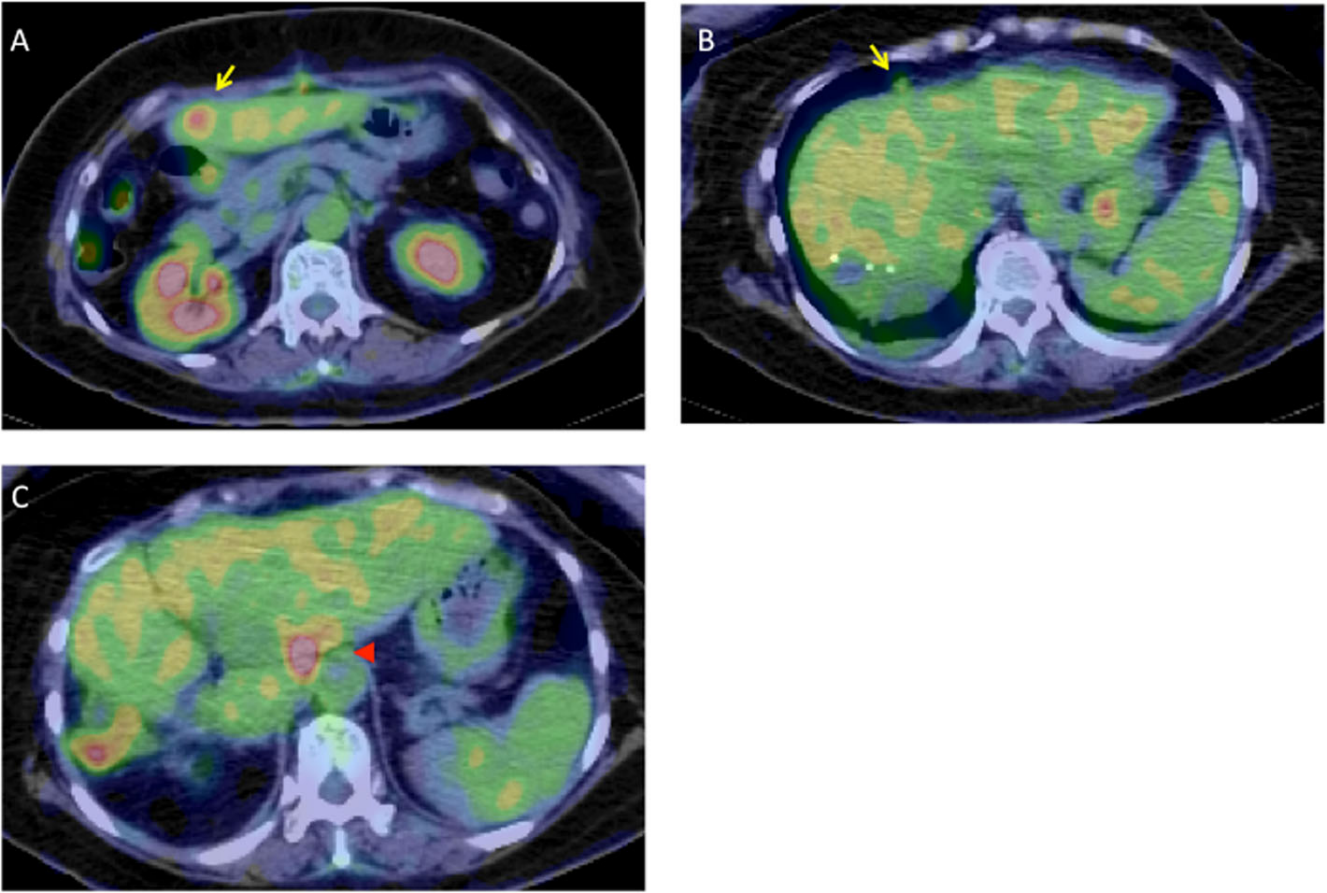
Positron emission tomography (PET) findings on POW 7. **a** PET showing intrahepatic recurrence (arrow). **b** PET showing peritoneal dissemination (arrow). **c** PET showing hilar lymph node metastasis (arrowhead)

After the surgery, the patient’s general condition was good and no neurological changes were observed. The perioperative changes in serum tumor maker levels are shown in Fig. 4. Serum CA19-9 remained above detectable levels until 4 days after surgery and decreased to 43,068 U/mL on postoperative day 4. By imaging, the splenic and renal infarcts were slightly enlarged and a new subacute subcortical hemorrhage in the right occipital lobe was observed on postoperative day 7. Starting at 1 week after surgery, anticoagulant therapy was resumed by continuous intravenous infusion of unfractionated heparin at a dose of 15,000 U per day. At 1-month postoperative evaluation, no new neurological changes were observed and there were no exacerbations in the infarcts and hemorrhagic lesions. The serum levels of CA19-9 started to increase, reaching 9459.4 and 80,256.2 U/mL at 4 and 8 weeks after the surgery, respectively. Although enhanced CT did not show recurrence, positron emission tomography suggested intrahepatic recurrence, hilar lymph node metastasis, and peritoneal dissemination at 7 weeks after the surgery (Fig. 5). Therefore, the patient was initiated on combination chemotherapy with gemcitabine and cisplatin at 8 weeks after the surgery. The chemotherapy regimen comprised cisplatin (25 mg/m^2^ body surface area) followed by gemcitabine (1000 mg/m^2^ body surface area) [6], both administered on days 1 and 8, every 3 weeks. This treatment was effective in stabilizing CA19-9 levels (Fig. 4) but did not cure the tumor recurrence.

Continuous intravenous heparin administration was changed to subcutaneous injection of low-molecular-weight heparin at a dose of 5000 U per 12 h, and she was discharged 12 weeks after the surgery. She has been subsequently followed in our outpatient clinic without further thrombosis during the last 6 months after surgery, and there has been no tumor progression.

## Discussion

Tumors associated with Trousseau’s syndrome are usually mucin-producing adenocarcinomas derived from visceral organs. However, few studies have reported an association between cholangiocarcinoma and thromboembolism [3, 7, 8]. The prognosis is extremely poor in patients with Trousseau’s syndrome related to primary cholangiocarcinoma [3, 7, 8], which was first reported by Ching in 1991 [7]. Eleven cases of Trousseau’s syndrome developed in intrahepatic cholangiocarcinoma have been reported in English [3, 7, 8]. Only three patients of eleven survived for more than 3 months, and there was no patient who underwent liver resection. However, the current patient underwent right hepatectomy and received systemic chemotherapy; she was successfully discharged and has been followed without further thrombosis during the 6 months after surgery.

Because most patients of Trousseau’s syndrome and intrahepatic cholangiocarcinoma are at an advanced stage by the time of diagnosis, it is difficult for them to undergo curative treatment [2, 3, 7, 9]. Therefore, the prognosis of the patients with Trousseau’s syndrome and intrahepatic cholangiocarcinoma is very poor [3, 7, 8]. Because preoperative evaluation suggested that curative resection was possible in this case, we chose surgical resection for the treatment of the original tumor under the anti-coagulation prophylaxis, even though there was a possibility of further episodes of thrombosis. This strategy was fully explained to the patient and the family, and they completely agreed with our principle. In fact, Moustafa et al. recommended liver resection to intrahepatic cholangiocarinoma patients in stage III and IVa without multifocal lesions [10].

In the present case, the serum levels of CA19-9 were above the detectable range until 4 days after the surgery. The reported half-life of CA19-9 immediately after surgery is 0.5 ± 0.1 days [11]. Therefore, the preoperative serum CA19-9 level was estimated to be over 1,000,000 U/mL in the present case. Given that tumor makers are usually considered only as diagnostic biomarkers, the pathological significance of an elevation in their levels is not seriously considered. However, the elevated tumor maker levels such as CA19-9, CEA, CA125, CA15-3, and Sialyl Lewis X (SLX) in the present patient might have been triggers for the observed thrombotic events (Table 1), as reported in a previous study [12]. Although thromboembolism in cancer patients is caused by multiple factors such as tissue factor, cytokines, and mucin, the tumor maker level should be kept as low as possible to avoid additional thrombotic events. Because Yuri et al. also reported a case of Trousseau’s syndrome accompanied with cholangiocarinoma with high CA19-9 [8], our case enforced their speculation about the causative factors to Trousseau’s syndrome. We should pay attention to thrombotic event in cholanginocarcinoma cases with high CA19-9. Although recurrence occurred just 7 weeks after the surgery in the present case, surgery might have been able to significantly reduce the CA19-9 levels. Immunohistochemistry showed an extremely strong expression of CA19-9. Even though the recurrence was not observed with conventional CT, likely due to the small size of liver masses, her CA19-9 was elevated a few weeks after surgery. Her cholangiocarcinoma was considered to produce extremely high levels of CA19-9.

Heparin is widely preferred over oral anticoagulants to prevent thrombotic events in patients with Trousseau’s syndrome [13]. In the present case, the infusion of unfractionated heparin was used as first-line treatment, and bridging anticoagulation with subcutaneous injection of low-molecular-weight heparin was performed during the perioperative period. Subsequently, the patient was switched to unfractionated heparin infusion followed by low-molecular-weight heparin in preparation for her discharge. The heparin treatment was successful, and the patient did not experience severe bleeding or thrombotic events in the perioperative period.

## Conclusion

In conclusion, we herein reported the rare case of a patient with cholangiocarcinoma who presented with Trousseau’s syndrome and successfully underwent right hepatectomy followed by systemic chemotherapy. As illustrated in the present case, an extremely high level of CA19-9 might trigger Trousseau’s syndrome in patients with cholangiocarcinoma.

## Data Availability

Not applicable

## Abbreviations

CT: Computed tomography
MRI: Magnetic resonance imaging
CA19-9: Carbohydrate antigen 19-9
CEA: Carcinoembryonic antigen
